# Supplementation of n-3 PUFAs in Adulthood Attenuated Susceptibility to Pentylenetetrazol Induced Epilepsy in Mice Fed with n-3 PUFAs Deficient Diet in Early Life

**DOI:** 10.3390/md21060354

**Published:** 2023-06-09

**Authors:** Ying-Cai Zhao, Cheng-Cheng Wang, Xiao-Yue Li, Dan-Dan Wang, Yu-Ming Wang, Chang-Hu Xue, Min Wen, Tian-Tian Zhang

**Affiliations:** 1 College of Food Science and Engineering, Ocean University of China, Qingdao 266404, China; zhaoyingcai@stu.ouc.edu.cn (Y.-C.Z.); wangccouc@163.com (C.-C.W.); lixiaoyue1278@stu.ouc.edu.cn (X.-Y.L.); wangdandan@stu.ouc.edu.cn (D.-D.W.); wangyuming@ouc.edu.cn (Y.-M.W.); xuech@ouc.edu.cn (C.-H.X.); 2 Institute of Biopharmaceutical Research, Liaocheng University, Liaocheng 252000, China; 3 Pet Nutrition Research and Development Center, Gambol Pet Group Co., Ltd., Liaocheng 252000, China

**Keywords:** early life nutrition, dietary supplementation, epilepsy, n-3 PUFAs deficiency, DHA-enriched phospholipids

## Abstract

The growth and development of the fetus and newborn throughout pregnancy and lactation are directly related to the nutritional status of the mother, which has a significant impact on the health of the offspring. The purpose of this experiment was to investigate the susceptibility of n-3 polyunsaturated fatty acid deficiency in early life to seizures in adulthood. The n-3 PUFAs-deficient mice’s offspring were established and then fed with α-LNA diet, DHA-enriched ethyl ester, and DHA-enriched phospholipid-containing diets for 17 days at the age of eight weeks. During this period, animals received intraperitoneal injections of 35 mg/kg of pentylenetetrazol (PTZ) every other day for eight days. The results showed that dietary n-3 PUFA-deficiency in early life could aggravate PTZ-induced epileptic seizures and brain disorders. Notably, nutritional supplementation with n-3 PUFAs in adulthood for 17 days could significantly recover the brain n-3 fatty acid and alleviate the epilepsy susceptibility as well as raise seizure threshold to different levels by mediating the neurotransmitter disturbance and mitochondria-dependent apoptosis, demyelination, and neuroinflammation status of the hippocampus. DHA-enriched phospholipid possessed a superior effect on alleviating the seizure compared to α-LNA and DHA-enriched ethyl ester. Dietary n-3 PUFA deficiency in early life increases the susceptibility to PTZ-induced epilepsy in adult offspring, and nutritional supplementation with n-3 PUFAs enhances the tolerance to the epileptic seizure.

## 1. Introduction

As an emblematic brain disease, epileptic seizures demonstrate dysregulated brain function. The two characteristics of epilepsy activities are action potential bursts, a type of neuronal discharge, and neuronal synchronization, a type of physiological group activity [1]. Epileptic activities involved in neuronal function homeostasis include ion channels and neurotransmitter receptors, the cellular and synaptic properties of neurons, and the collective activities of groups of interconnected neurons modulated by glial cells [2]. Worldwide, approximately 50 million individuals are currently suffering from epilepsy, which accounts for 1% of the global burden [3,4]. Early-life nutrition manipulated brain development and functional characteristics, and congenital nutritional status could affect organ development and epilepsy.

Omega-3 polyunsaturated fatty acids (n-3 PUFAs) are essential for the functional maturation of the brain, and low maternal consumption of n-3 PUFAs has been associated with neurodevelopmental disorders in offspring [5]. Evidence suggests that inadequate levels of n-3 PUFAs may represent a risk factor for these disorders. Wang et al. reported that dietary n-3 PUFA-deficiency in early life exhibited more aggravated 1-methyl-4-phenyl-1,2,3,6-tetrahydropyridine (MPTP)-induced neurotoxicity and increased vulnerability to scopolamine-induced cognitive impairment in male offspring [6,7]. Furthermore, maternal dietary n-3 PUFA deficiency increases microglia-mediated phagocytosis of synaptic elements in the rodent developing hippocampus, partly through the activation of 12/15-lipoxygenase (LOX)/12-hydroxyeicosatetraenoic acid (HETE) signaling, altering neuronal morphology and affecting the cognitive performance of the offspring [8]. Besides, the n-3 PUFAs have the properties of cellular electrical signal homeostasis, which are known to have antiarrhythmic effects and appear to be mediated by their actions on voltage-dependent sodium channels [9]. Nabekura et al. demonstrated that n-3 PUFAs reduced sodium currents and increased voltage-dependent sodium channel (VDSC) inactivation time [10]. It has been reported that adequate docosahexaenoic acid (DHA) availability in the fetus optimizes brain and retinal maturation in part by influencing neurotransmitter pathways [11]. A variety of actions of DHA on the activity of γ-aminobutyric acid A (GABAA) receptor channel complex emphasizes the importance of the lipid microenvironment for the activity of ligand-gated ionic channels [12].

Epilepsy caused by abnormal discharge of nerve cells can be improved by the regulation function of n-3 PUFA supplementation. Clinical evidence showed that eicosapentaenoic acid (EPA) and DHA administration had alleviation effects on seizure frequency in patients with drug-resistant epilepsy [13], and DHA could reduce susceptibility to febrile seizures owing to the increase of estrogen contents in the brain [14]. Maternal dietary deficiency of n-3 PUFAs could result in a decrease in n-3 fatty acids in the brains of offspring. Previously, Sanjay et al. reported that a maternal dietary n-3 PUFA deficiency altered neurotransmission in the developing hippocampus [15]. Therefore, we speculated that the epileptic seizure might be aggravated by the decline of n-3 PUFAs in the brain. Moreover, it is still unclear whether the supplementation of n-3 PUFAs in adulthood could attenuate the susceptibility to epilepsy in the case of dietary n-3 PUFA deficiency in early life.

To determine the effect of maternal dietary n-3 PUFA deficiency on the epileptic seizure severity of the offspring, we investigated the antiepileptic effects of n-3 PUFAs as well as their role in the amelioration of neurological deficits by utilizing a n-3 PUFA deficiency mouse model of pentylenetetrazol (PTZ)-induced epilepsy. It was found that the offspring of maternal n-3 PUFA deficiency exhibited severe convulsant activity and neurological deficits, and supplementation with the n-3 PUFAs could ameliorate the seizure via multiple signaling pathways. Our findings identify imbalanced nutrition as a potent environmental risk factor for the neurodevelopmental disorders and provide a basis for antiepileptic effects of n-3 PUFAs.

## 2. Results

### 2.1. Effects of Dietary n-3 PUFAs in Adulthood on PTZ-Induced Epileptic Seizure of Mice Feeding n-3 PUFAs Deficiency in Early Life

**Figure 1:**
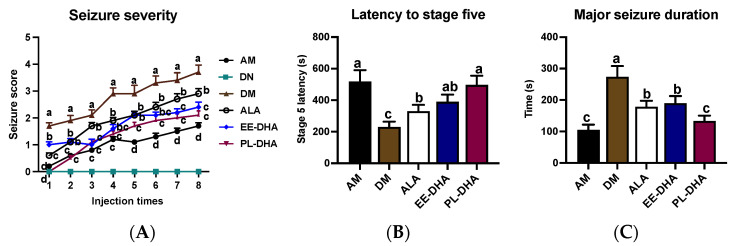
Seizure parameters of each group after injections of 35 mg/kg·bw PTZ every other day. Seizure intensity scores and latency to stage V, as well as the major seizure duration, were assessed after PTZ administration. (**A**) The variation of seizure severity in mice after intraperitoneal injection of PTZ every other day. (**B**) The latency to major seizure of stage V after intraperitoneal injection of PTZ every other day. (**C**) Major seizure duration of the mice after PTZ injection. The data were expressed as mean + SEM (*n* = 10). One-way ANOVA was utilized to evaluate the difference among the groups, and different letters indicated significant differences determined by Tukey’s test.

In this experiment, no differences were observed in terms of body parameters or growth rate between the n-3 PUFAs-deficient mice and the n-3 PUFAs-adequate mice (Figure S1). Intraperitoneal injection of continuous subconvulsive doses of PTZ, a GABAA receptor antagonist, into animals results in a model of persistent and severe epilepsy. Regarding the seizure behavior, compared with the PTZ-treated n-3 PUFAs-adequate group, the n-3 PUFAs deficient group aggravated seizure progression (Figure 1A) and reduced seizure latency to stage five by 55.7% (Figure 1B), as well as prolonged the duration of major seizures by 1.6-fold (Figure 1C). This result demonstrated that n-3 PUFA deficiency in early life caused increased susceptibility to epileptic seizures in adulthood.

Further comparison revealed that supplementation with n-3 PUFAs in adulthood could alleviate the increased susceptibility to epilepsy caused by early-life n-3 fatty acid deprivation. Interestingly, different types of dietary n-3PUFAs in adulthood for 17 days exhibited different degrees of seizures after PTZ administration in mice. Results showed that α-LNA (ALA), DHA-enriched ethyl ester, and DHA-enriched phospholipid administrations significantly increased the latency to stage five from 230 s to 330 s, 394 s, and 497 s, and decreased the major seizure duration from 273 s to 178 s, 190 s, and 134 s, respectively, reflecting the different anticonvulsant effect of n-3 PUFAs against PTZ-induced seizure. Data suggested that deprivation of n-3 PUFAs early in life exacerbated susceptibility to epilepsy in adulthood, while dietary different types of n-3 PUFAs in adulthood could alleviate epileptic seizure with different degrees, in which DHA-enriched phospholipids exhibited significant effects compared with α-linolenic acid and DHA-enriched ethyl ester.

### 2.2. Effects of Dietary n-3 PUFAs in Adulthood on Neurotransmitter Disturbances Associated with Epilepsy in Mice with Early-Life n-3 PUFAs Deficiency

**Figure 2:**
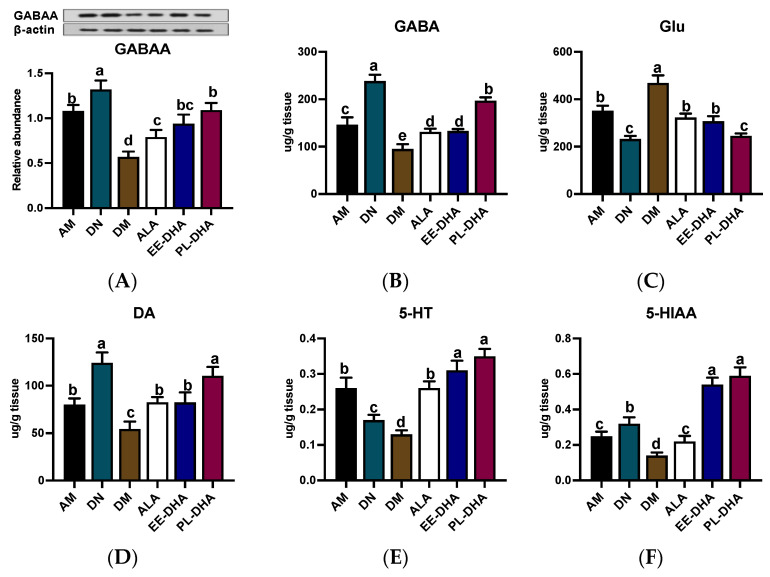
The concentrations of GABAA receptor and monoamine neurotransmitter as well as the metabolites in the hippocampus after PTZ intraperitoneal injection (*n* = 7). (**A**) Representative Western blot bands and quantitative analysis of hippocampal expression of GABAA receptor densitometric values of the signals were quantified and expressed as the ratio to β-actin. Monoamine neurotransmitter content of (**B**) GABA, (**C**) Glu, (**D**) DA, (**E**) 5-HT, and (**F**) 5-HIAA in the hippocampus. Different letters among the groups represented significant differences determined by one-way ANOVA (Tukey’s test). γ-aminobutyric acid (GABA), glutamate (Glu), 5-hydroxytryptamine (5-HT), and 5-hydroxyindoleacetic acid (5-HIAA).

In PTZ-induced epilepsy, the level of γ-aminobutyric acid (GABA) with presynaptic inhibitory function is significantly decreased, and meanwhile glutamate (Glu) is highly active, which mainly acts as an excitotoxic, postsynaptic excitatory neurotransmitter [16]. For GABAA receptor expression, PTZ injection resulted in a decrease in GABAA receptor expression, which was further exacerbated by n-3 PUFA deficiency in early life but was restored to varying degrees by different types of n-3 PUFAs in adulthood (Figure 2A). As is shown in Figure 2B,C, administration of PTZ resulted in a significant decrease in GABA and a dramatic increase in Glu compared to the n-3 PUFA-deficient control group (DN). Notably, GABA abundance in the n-3 PUFA deficient group treated with PTZ (DM) was 54.3% lower than that in the n-3 adequate group (AM) group, while Glu was 33.6% higher in the DM group than that in the AM group. Supplementation with different types of n-3 PUFAs in adulthood for 17 days was effective in reducing Glu and GABA imbalances induced by PTZ injection, and the effect of DHA-enriched phospholipid was significant compared to DHA-enriched ethyl ester and α-LNA.

Moreover, the generation, aberrant release, and inactivation of monoamine neurotransmitters such as dopamine (DA) and 5-hydroxytryptamine (5-HT) in the brain could cause abnormal discharge of neurons with high synchronization and induce epilepsy. Obviously, n-3 PUFAs-deficient mice exhibited lower levels of DA (54.4 ug/g tissue) and 5-HT (130.1 ng/g tissue) after PTZ exposure compared to the n-3 PUFAs-adequate group (AM) (Figure 2D,E). Significant recovery of DA and 5-HT was noted when different types of n-3 PUFAs were administered to mice exposed to PTZ in adulthood, although DA levels in the DHA-enriched ethyl ester and α-LNA treated groups remained significantly lower than those observed in n-3 PUFAs-deficient controls (DN). Interestingly, DHA phospholipid supplementation was able to restore DA and 5-HT in the DM group to levels comparable to those in the DN group. Moreover, 5-HT and 5-hydroxyindole-3-acetic acid (5-HIAA) in the groups receiving DHA-enriched ethyl ester and DHA-enriched phospholipid recovered to DN group levels, although administration of α-LNA to PTZ-exposed mice did not restore levels of 5-HIAA, a metabolite of 5-HT, comparable to those in the control group (DN) (Figure 2F).

### 2.3. Effects of Dietary n-3 PUFAs in Adulthood on the Central Nervous System Injury and Neurogenesis in Mice with Early-Life n-3 PUFAs Deficiency and PTZ-Induced Epilepsy

**Figure 3:**
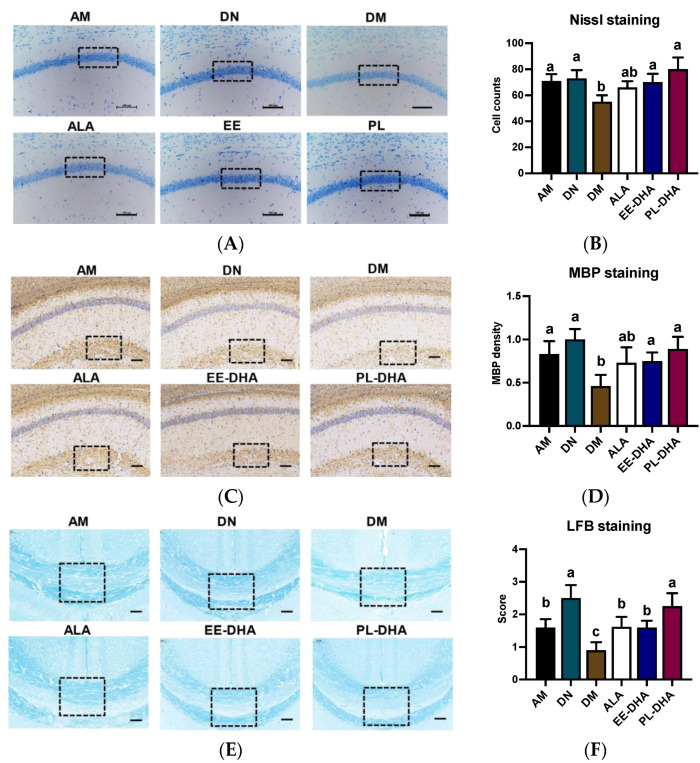
Effects of brain n-3PUFA levels on PTZ-induced neuron loss and demyelination in the central nervous system. (**A**) Nissl histochemical stain for Nissl bodies in the neurons of the CA1 region in the hippocampus (×400 magnification, scale bar = 100 μm). (**B**) Nissl body-positive neurons in selected CA1 regions of the hippocampus. Data were calculated at three random positions in the CA1 area in each section according to the number of nuclei stained by Nissl staining (*n* = 3). (**C**) Immunohistochemical staining was performed to evaluate the expression levels of MBP in hippocampus (×400 magnification, scale bar = 100 μm). (**D**) Expression levels of MBP in the groups. The average pixel density of MBP staining in three random positions of the CA1 region in each slice was used to evaluate the MBP expression among the groups (*n* = 3). The results of MBP density are expressed in arbitrary units. (**E**) Evaluation of myelin content with Luxol Fast Blue (LFB) staining after PTZ exposure. (**F**) Semiquantitative demyelination scores of LFB staining in each section were assessed by a semiquantitative four-tried scoring system: Intact myelin was scored with 0, and the totally damaged myelin was labeled with a score of 3 (*n* = 3). Dashed dimension areas in (**A**,**C**,**E**) represented typical differences in slices of the groups. Different letters among the groups represented significant differences determined by one-way ANOVA (Tukey’s test).

Nissl staining was performed to identify the neuronal damage, and results showed that the number of nissl bodies in the hippocampal CA1 region of n-3 PUFAs-deficient mice administered with PTZ was significantly reduced compared to mice in the n-3 PUFAs-adequate group (AM). In the n-3 PUFA-supplemented groups, DHA-enriched ethyl ester and DHA-enriched phospholipid treatment restored the nissl bodies to the level of the n-3 PUFA-adequate group (AM). These results demonstrated that there was apparent neuronal damage in the hippocampus of n-3 PUFA-deficient mice induced by PTZ, and that the damaged neurons could be restored after n-3 PUFA supplementation in adulthood (Figure 3A,B).

To further demonstrate PTZ-induced defects in the central nervous system (CNS), we performed luxol fast blue (LFB) staining and myelin basic protein (MBP) staining, a major constituent of CNS myelin and mature oligodendrocytes for myelin, on paraffin-embedded coronal sections. The results showed that there was a diminished staining of MBP in the brain of the n-3 PUFA-deficient mice after PTZ treatment (DM), as depicted by the staining intensity of MBP. Conversely, a restoration in MBP density was observed after different types of n-3 PUFA supplementation in adulthood (Figure 3C,D). Overall staining intensity of MBP in mice was markedly restored in the ALA, EE-DHA, and PL-DHA groups as compared to the DM group. Patterns of LFB in the brain were similar to the MBP intensity in mice (Figure 3E,F). The results showed that dietary n-3 PUFAs deficient in early life and adulthood showed severe demyelination in the brain after PTZ injection, compared to a diet that was adequate in n-3 PUFAs.

**Figure 4:**
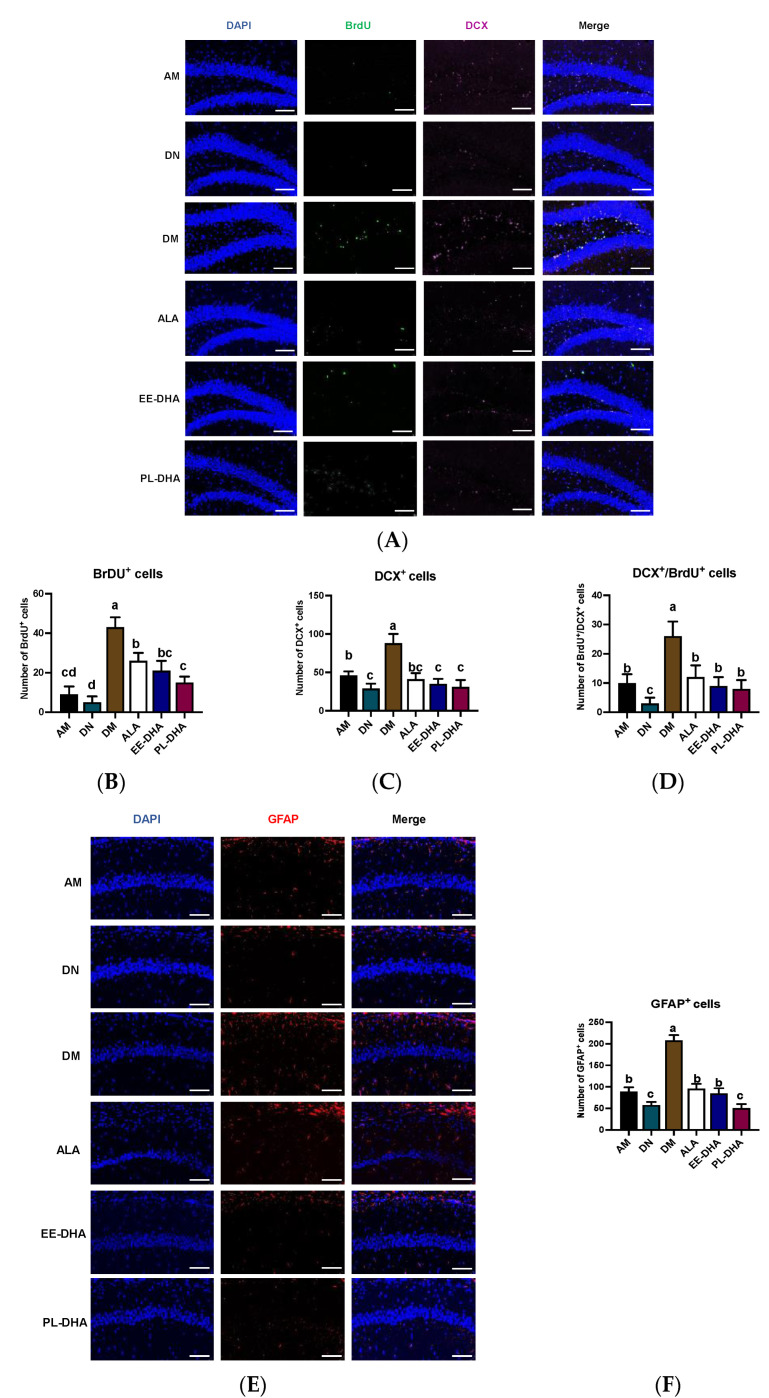
Effects of brain n-3 PUFA levels on PTZ-induced neurogenesis in the CA1 region of the hippocampus. (**A**) Representative image from immunofluorescence staining of DAPI (Blue) and BrdU (Green) and DCX (Pink) in hippocampal DG region of the PTZ administrated mice. Scale bar = 100 µm. The number of BrdU+ neurons (**B**), DCX+ neurons (**C**), and BrdU+ DCX+ neurons (**D**) in each slice. Effects of brain n-3 PUFA levels on GFAP expression in CA1 region of hippocampus. (**E**) Representative image from immunofluorescence staining of DAPI (Blue) and GFAP (Red) in hippocampus of the PTZ treated mice. Scale bar = 100 µm. (**F**) The number of GFAP+ cells in CA1 region of the hippocampus in each slice. Data are reported as mean ± SEM (*n* = 3), and different letters among the groups represented significant differences determined by one-way ANOVA (Tukey’s test).

Apart from the injury to the CNS, PTZ-induced seizures could affect neurogenesis. The immune fluorescence of bromo-deoxyuridine (BrdU) and doublecortin (DCX, an immature neuronal marker) paradigm reflects a combination of both neuron proliferation and subsequent survival at the time of sacrifice. Using double immunohistochemistry with DCX and BrdU (Figure 4), we demonstrated that the total number of immature neurons and neuronal-oriented precursors double-labeled with BrdU and DCX was affected by dietary n-3 PUFA levels in early life following PTZ administration. As shown in Figure 4A, the number of BrdU-positive cells was significantly increased in n-3 PUFAs-deficient mice compared with n-3 PUFAs-adequate mice after PTZ injection to induce epilepsy, and supplementation with different types of n-3 PUFAs in adulthood could significantly reduce the incorporation of BrdU in the hippocampal dentate gyrus (DG) region. Interestingly, the expression of DCX in the hippocampal DG region was nearly consistent with BrdU, and neurogenesis was correlated with total n-3 PUFA levels in the mouse brain. Furthermore, the majority of BrdU-positive cells in the hippocampus were double-labeled by DCX and BrdU, suggesting that PTZ-induced seizures affect neurogenesis (Figure 4B–D). As for the marker of astrocytes, the number of glial fibrillary acidic protein (GFAP)-positive cells in the CA1 region of the hippocampus after PTZ stimulation was significantly increased in the DM group compared with the AM group, implying that dietary n-3 PUFA deficiency aggravated the PTZ-induced activation of astrocytes effectively (Figure 4E,F). Supplementation with n-3 PUFA-enriched diets could decrease the hyperactivation of astrocytes. These results illustrated that the PTZ-induced abnormal neurogenesis in the mice fed with a n-3 PUFA-deficient diet in early life could be improved by different types of n-3 PUFA administration in adulthood, of which DHA-enriched phospholipid exhibited the most noticeable effect.

### 2.4. Effects of Dietary n-3 PUFAs in Adulthood on AKT/CREB/BDNF Pathway and Synaptic Plasticity in Mice with Early-Life n-3 PUFAs Deficiency and PTZ-Induced Epilepsy

**Figure 5:**
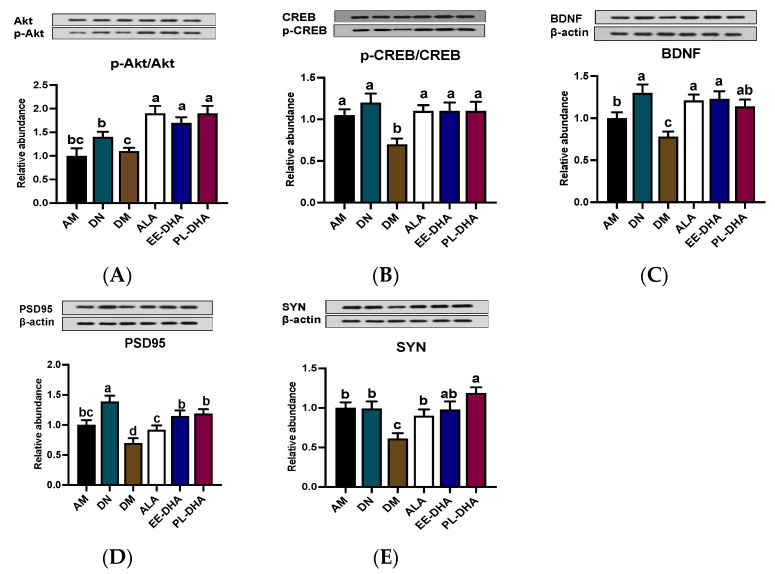
Effects of brain n-3 PUFA levels on the AKT/CREB/BDNF pathway and synaptic plasticity related gene expression in PTZ-induced seizure mice. (**A**–**E**) Representative Western blot bands and quantitative analysis of hippocampal expression of p-Akt/Akt, p-CREB/CREB, BDNF, PSD95, and SYN levels through densitometry analysis of western blot graphs. Densitometric values of the signals were quantified and expressed as the ratio to β-actin. Data are reported as mean ± SEM (*n* = 7). Different letters indicated significant differences (*p* < 0.05) in the six groups. Densitometry of relative band intensity was determined using Image J software.

Inhibiting neuronal apoptosis to prevent epilepsy appears to be possible with the activation of the AKT/CREB/BDNF signaling pathway. The phosphorylation levels of AKT and CREB in the DM group showed a significant decline compared with those of the AM group, while supplementation with n-3 PUFAs could increase the phosphorylation levels of AKT and CREB. Notably, there was no significant difference among the ALA, EE-DHA, and PL-DHA groups (Figure 5A,B). The expression of BDNF in the six groups showed similarity to the expression trend of its upstream (Figure 5C). Moreover, DHA-enriched ethyl ester and DHA-enriched phospholipid supplementation for 17 days remarkably promoted the synaptic plasticity-related proteins PSD95 and SYN expression (Figure 5D,E).

### 2.5. Effects of Dietary n-3 PUFAs in Adulthood on Mitochondria-Dependent Apoptosis in the Hippocampus of PTZ-Induced Epileptic Mice with Early-Life n-3 PUFAs Deficiency

**Figure 6:**
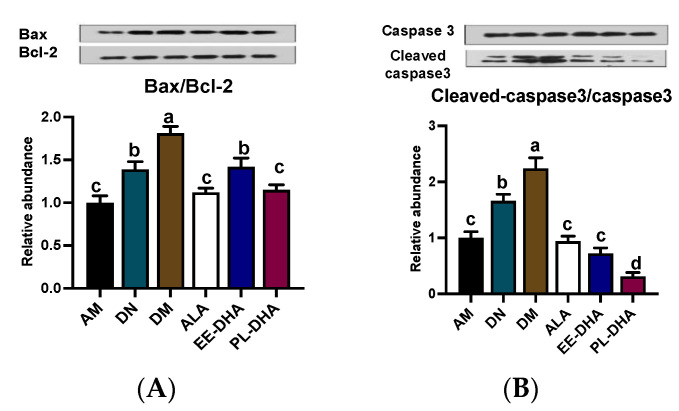
Effects of the brain n-3 PUFA levels on the expression of mitochondria-dependent apoptosis pathway related gene expression in PTZ-induced seizure mice. Representative Western blot band and quantitative analysis of hippocampal expression of Bax/Bcl-2 (**A**) and Cleaved-caspase3/Caspase3 (**B**) levels through densitometry analysis of western blot graphs. Densitometric values of the signals were quantified and expressed as the ratio to β-actin. Data are reported as mean ± SEM (*n* = 7). Different letters indicated significant differences (*p* < 0.05) in the six groups. Densitometry of relative band intensity was determined using Image J software.

PTZ-induced epilepsy could lead to mitochondrial lesion, and further promote the development of hippocampus neuron apoptosis. In this study, compared to the DN group, PTZ administration significantly aggravated the mitochondria-dependent apoptosis degree. The mice in n-3 PUFAs deficient group (DM) showed a higher ratio of Bax/Bcl-2 by inhibiting the anti-apoptotic Bcl-2 and activating the pro-apoptotic Bax expressions, and a higher ratio of cleaved-Caspase-3/Caspase-3 by activating the pro-apoptotic cleaved-caspase-3 expression than the AM group. Notably, n-3 PUFAs supplementation downregulated pro-apoptotic protein expression and improved mitochondria-dependent apoptosis. In detail, both the ratios of Bax/Bcl-2 and cleaved Caspase-3/Caspase-3 in the three n-3 PUFAs supplemented group mice recovered to the n-3 PUFAs adequate control level (AM) (Figure 6A,B).

### 2.6. Effects of Dietary n-3 PUFAs in Adulthood on Hippocampal Neuroinflammation in PTZ-Induced Epileptic Mice with Early-Life n-3 PUFAs Deficiency

**Figure 7:**
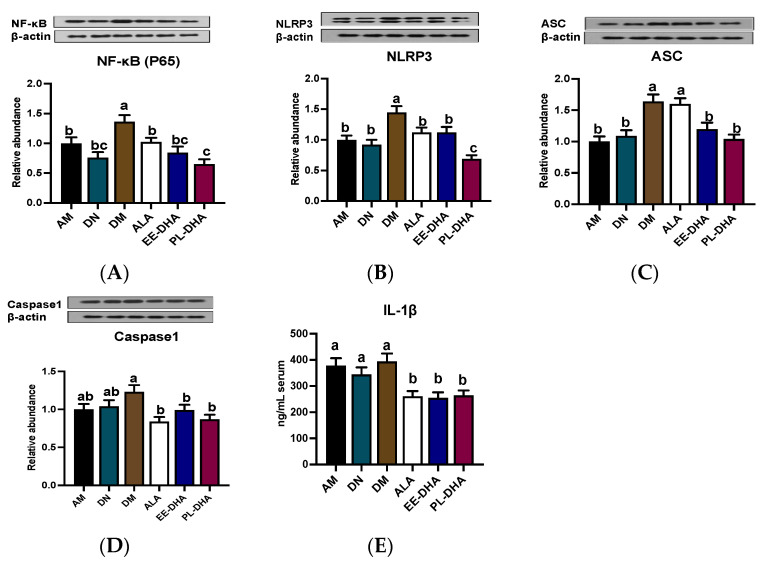
Effect of different n-3 PUFA statuses in early life and adulthood on the NLRP3 inflammasome pathway in the hippocampus. (**A**–**D**) Representative Western blot bands and quantitative analysis of hippocampal expression of NF-κB (P65), NLRP3, ASC, and caspase1 after PTZ treatment through densitometry analysis of Western blot graphs. Densitometric values of the signals were quantified and expressed as the ratio to β-actin. Data are reported as mean ± SEM (*n* = 7). (**E**) The content of IL1-β in serum after PTZ administration (*n* = 7). Different letters indicated significant differences (*p* < 0.05) in the six groups. Densitometry of relative band intensity was determined using Image J software.

The NLPR3-inflammasome, composed of the NLRP3 scaffold, apoptotic speck-containing protein (ASC) linker, caspase1, and the upstream NF-κB (P65), has been implicated in central nervous system (CNS) inflammatory diseases. As is shown in Figure 7, enhanced expression of NF-κB (P65), NLRP3, ASC, and caspase1 was observed in the n-3 PUFA-deficient group (DM) compared to the n-3 PUFA-adequate group (AM), suggesting that dietary n-3 PUFA deficiency aggravated the PTZ-induced activation of neuroinflammation. Moreover, supplementation with different types of n-3 PUFAs in adulthood could reduce protein expression to varying degrees, of which DHA-enriched phospholipids exhibited a noticeable effect in restoring the protein expression of NF-κB (P65) and NLRP3 (Figure 7A,B). It should be noted that supplementation with n-3 PUFAs had a similar regulatory effect on the expression of ASC and caspase1 (Figure 7C,D). However, the effect of α-LNA on the reduction of ASC expression was not comparable to that of DHA-enriched ethyl ester and DHA-enriched phospholipid. Besides, there was no significant difference among the three n-3 PUFA-supplemented groups in the regulation of caspase1. IL-1β, as the downstream of NLRP3 pathway, was increased in PTZ treated n-3 PUFAs deficiency group (DM) compared with that of the n-3 PUFA-adequate group (AM). Mice lacking n-3 PUFAs in early life supplemented with n-3 PUFAs in adulthood could reduce IL-1β levels, although the effect differed little among different forms of n-3 PUFAs (Figure 7E).

### 2.7. Effects of Dietary n-3 PUFAs in Adulthood on Hippocampal Fatty Acid Composition of PTZ-Induced Epileptic Mice with Early-Life n-3 PUFAs Deficiency

As shown in Table 1, DHA levels in the hippocampus of n-3 PUFA-deprived mice were reduced by more than 40% (*p* < 0.05), indicating that chronic n-3 PUFA-deficient diets significantly reduced brain DHA levels in mice. Compared with the saline-treated n-3 PUFAs deficient group (DN), PZT injection did not result in decreased brain DHA levels in the DM group. In addition, after supplementation with different types of n-3 PUFAs in adulthood, the brain DHA levels of n-3 PUFAs-deficient mice were significantly changed compared with those of n-3-deficient groups (DN group and DM group), and were increased by 49%, 73%, and 84% after α-LNA, DHA-enriched ethyl ester and DHA-enriched phospholipid treatments, respectively, compared to those of the DM group. Interestingly, supplementation with DHA-enriched ethyl ester and DHA-enriched phospholipid for 17 days almost recovered to levels comparable to the AM group, while α-LNA supplementation did not achieve this effect. The results suggested that DHA-enriched ethyl ester and DHA-enriched phospholipid were more effective in restoring brain DHA than α-LNA.

Regarding n-6 PUFAs, hippocampal arachidonic acid (ARA, 20:4n-6) and docosapentaenoic acid (DPA, 22:5n-6) levels in the dietary n-3 PUFA-deficient group were significantly increased (*p* < 0.05), and docosatetraenoic acid (DTA, 22:4n-6) was not significantly changed, compared with the AM group. After supplementation with different types of n-3 PUFAs for 17 days in adulthood, the n-6/n-3 ratios of the ALA, EE-DHA, and PL-DHA groups were significantly decreased (*p* < 0.05). In addition, there were no significant differences in total saturated fatty acid, monounsaturated fatty acid, or polyunsaturated fatty acid content among all dietary intervention groups.

## 3. Discussion

Malnutrition during pregnancy and lactation may result in adaptive regulation of fetal development, making adult offspring respond differently to external environmental stimuli and diseases. In this work, we found that maternal dietary n-3 PUFA deficiency resulted in increased susceptibility of offspring to PTZ-induced epilepsy. Supplementation with different types of n-3 PUFAs in adulthood reduced epilepsy severity and latency to varying degrees. Further analysis revealed that n-3 PUFA supplementation was able to modulate epilepsy-induced neurotransmitter imbalance, reduce inflammation, neuronal damage, demyelination, and apoptosis. The findings revealed that a decline in n-3 PUFAs in the brain contributed to PTZ-induced severe brain dysfunction, while supplementation with n-3 PUFAs ameliorated the damage and alleviated brain disorders.

DHA, a representative n-3 polyunsaturated fatty acid in the brain, has been recognized as a molecule with functional properties to promote brain development, maintain central nervous system function, and have anti-inflammatory activity [17]. Previous studies reported that DHA had functional properties for regulating abnormal electrical signals as well as antiarrhythmic effects [18]. Trépanier et al. reported that DHA significantly increased seizure latency by approximately 3-fold as compared to vehicle-injected animals [19]. Our study demonstrated that dietary deficiency of n-3 PUFAs in early life exacerbated the susceptibility to PTZ-induced seizures in adulthood, while DHA administration for about two weeks in adulthood alleviated the seizures and brain dysfunction. Anticonvulsant effects have been similarly reported in several studies involving short-term parenteral DHA administration in humans or rats [20,21].

The type and structure of n-3 PUFAs are important factors affecting their function. The major n-3 PUFA in flaxseed oil is α-LNA, while DHA is the major n-3 PUFA in DHA-enriched ethyl ester and DHA-enriched phospholipid extracted from squid roes. α-LNA is a precursor substance of EPA and DHA, which converts into EPA and DHA under the action of desaturase at a low productivity [22]. Interestingly, in the presence of reduced n-3 PUFAs in the brain, the overall improvement of PTZ-induced epilepsy by DHA-enriched phospholipid was superior to that of DHA-enriched ethyl ester and α-LNA. This may be related to the different digestion and absorption of DHA-enriched phospholipids and other forms of n-3 PUFAs. Moreover, as an important component of the nerve cell membrane, the structural properties of DHA-enriched phospholipids have advantages in maintaining the homeostasis of the CNS [23].

The mechanism proposed for seizures is the disruption of synaptic excitation and inhibition balance, which promotes neuronal hyperexcitability and hyper synchronization, through either an increase in excitatory synaptic transmission or a decrease in inhibitory synaptic transmission or both [24,25]. As an antagonist of GABAA receptors, intraperitoneal injection of PTZ can cause persistent convulsions, synaptic dysfunction, and nerve signal transmission disorders manifested as increased excitatory neurotransmitters and decreased inhibitory neurotransmitters [26]. The deficiency of n-3 PUFAs in the brain of epilepsy model mice is manifested as decreased GABA, 5-HT, and 5-HIAA content, excessive hippocampal Glu content, and decreased GABAA receptor expression, which were restored after n-3 PUFA supplementation by stabilizing the hyperexcitability neurons and neurotransmission. A previous study reported that depression is the most common comorbidity in patients with epilepsy [27]. DA, the neurotransmitter strongly linked to depression, exhibited an approximate change. Furthermore, manipulations that enhance the stability of neurotransmission, especially GABA and Glu, might be anti-epileptic and therapeutically advantageous.

The pathogenesis of epilepsy is complex and closely related to the imbalance of neurotransmitter effects, abnormal ion channels, neural network remodeling, and intracranial inflammatory activation. PTZ administration could stimulate the mitochondria of the neuron in the hippocampus to initiate programmed cell death (apoptosis). Previous studies observed a mass of apoptotic neurons in the brains of patients with seizures [28], indicating apoptosis was involved in the degeneration and deletion of hippocampal neurons. N-3 PUFAs, especially DHA and EPA, have been reported to have beneficial effects against brain oxidative damage and inhibit apoptosis by downregulating Bax/Bcl-2 expression and inhibiting caspase3 activation [29]. The present study showed that n-3 PUFA deficiency in the brain significantly exacerbated the extent of PTZ-induced apoptosis, which was consistent with previous studies. Besides, existing experimental evidence found that dentate gyrus progenitor cell proliferation was associated with status epilepticus-induced neuronal injury [30]. The immunohistochemistry demonstrated significant neurogenesis classified as neurons and astrocytes in hippocampus of n-3 PUFA-deficient mice with PTZ administration. As an important inflammasome, the activation of NLPR3 could promote the release of inflammatory effectors and aggravates epileptic seizures. Moreover, NF-κB (P65) signaling would be activated, leading to the upregulated transcription of inflammasome-related components, such as NLRP3, pro-IL-1, and pro-IL-18 in epilepsy. The inflammasome-adaptor protein ASC is recruited to NLRP3, and interacts with caspase-1, leading to its activation, and activated caspase-1 catalyzes the maturation of pro-inflammatory cytokines [31]. Administration of DHA-enriched phospholipid for 17 days in adulthood alleviated the brain damage and restored the PTZ-induced epileptic mice with early-life n-3 PUFA deficiency to normal levels by down-regulating the activation of NLRP3, ASC, caspase1, and the NF-κB signaling pathway.

BDNF is involved in activating many intracellular signaling pathways of neurodevelopment, such as the mitogen-activated protein kinase (MAPK), phosphatidylinositol 3-kinase (PI3K)/AKT cascades, as well as the phosphorylation of CREB [32]. Dendritic spines are the main site of synaptic input in the central nervous system. Previous studies have shown that epilepsy could lead to dendritic spine loss and synaptic structural damage, affecting synaptic plasticity [33]. The enhanced expression of PSD-95 and synaptophysin (SYN) in n-3 PUFA supplementation groups compared with n-3 PUFA-deficient groups suggests that viable dendritic spines are functional for brain homeostasis in PTZ-induced epilepsy. Previous studies have shown that infant deprivation of n-3 PUFAs significantly reduced brain DHA levels and was accompanied by reduced BDNF expression and abnormal synaptic development and function [34], which was consistent with the results of the present study, suggesting that increased susceptibility to epilepsy results in impaired neurodevelopment. The above results of n-3 PUFA supplementation, in the aspect of neurodevelopment and synaptic plasticity certified the significant neuroprotective effect of n-3 PUFAs, especially DHA-enriched phospholipids. Furthermore, prior studies indicated that epilepsy might be associated with latent pathological changes associated with demyelination, resulting in brain function disturbances and the potential initiation of psychiatric disorders [35]. Myelination in the central nervous system is regulated by various factors through a complex network of signals. N-3 PUFAs are involved in myelination and have an important impact on brain function. The expression of MBP and the pathological changes of LFB demonstrated n-3 PUFA deficiency in early life, which exacerbated demyelination in PTZ-induced epilepticus state.

In addition, epigenetic alterations may be one of the molecular mechanisms by which n-3 PUFA levels in the early diet influence the different levels of brain damage induced by external environmental stimuli in adulthood. Previous studies reported that maternal n-3 PUFA deficiency altered uterine artery remodeling and the placental epigenome in mice [36]. We hypothesize that early nutritional n-3 PUFA deficiency may lead to the alteration of DNA methylation status in the mouse brain, thereby affecting resistance to seizures, but the exact mechanisms need to be further investigated. Furthermore, male mice were used in the present study since female mice were susceptible to estrous cycles and hormone levels. Sex differences in epilepsy susceptibility should be clarified in future studies.

## 4. Materials and Methods

### 4.1. Animals and Administration

**Figure 8:**
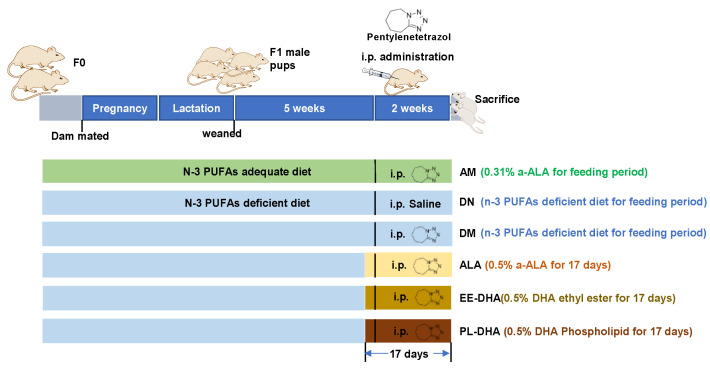
Experimental design and animal administration. After dietary intervention for three days, mice were intraperitoneally injected with pentetrazol (PTZ) every other day for a total of eight times.

All animal experimental procedures were performed according to the guidelines of the Ethical Committee of the College of Food Science and Engineering of the Ocean University of China (Approval No. SPXY2020012). Male and female ICR mice (weight 25–30 g, 8 weeks old) were purchased from Ji’nan Pengyue Laboratory Animal Breeding CO., Ltd. (Ji’nan, China) and housed at the SPF animal room under a temperature of 20 ± 2 °C and a humidity of 60% with a 12/12 h light/dark cycle (light starting at 8 a.m.). After 1 week of acclimatization, 18 male and 18 female mice (F0) were mated at the sex ratio of 1:1 and divided into two groups, in which six male and six female mice were fed with an n-3 PUFAs-adequate diet (containing 0.31% α-linolenic acid [α-LNA]), and the other mice were fed with an n-3 PUFAs-deficient diet modified from the standard AIN-93G diet. A single male and a single female were continuously housed together in a mouse cage from the mating period until the delivery of pups at postnatal days three to five. The pregnant dam continued to consume her assigned diets throughout gestation and lactation. The male offspring were weaned at 21 days of age and housed in groups of three to four animals per cage with food and water ad libitum. The offspring (F1) were kept on the same diet as their mothers (Figure 8). At eight weeks of age, male offspring fed an adequate n-PUFA diet were injected with 35 mg/kg·bw pentylenetetrazol (AM). The male offspring fed with a n-3 PUFA deficiency diet (α-LNA < 0.1%) were randomly assigned to five subgroups (*n* = 10 male mice per group), including one group injected intraperitoneally with saline (DN), and the other four groups were injected with 35 mg/kg·bw pentylenetetrazol (PTZ) and fed with n-3 PUFA-deficient diet (DM), α-LNA-enriched diet (ALA), a DHA-enriched ethyl ester-containing diet (EE-DHA), and a DHA-enriched phospholipids-containing diet (PL-DHA). The n-3 PUFA-enriched oil added to α-linolenic acid enriched diet was flaxseed oil. The oil added to the DHA-enriched ethyl ester-containing diet was ethyl ester fish oil, and for DHA-enriched phospholipid-containing diet was phospholipids extracted from squid roes. The n-3 PUFAs content in the ALA, EE-DHA, and PL-DHA groups was 0.5% *w*/*w* of the diet.

Mice were injected intraperitoneally with PTZ every other day for a total of 8 times to induce a developmental epilepsy model [37]. The latency, duration, and intensity of each epileptic seizure were recorded. Seizure intensity scores were graded on the Racine scale [38]: Grade V: generalized tonic-clonic seizures and falls; Grade IV: standing with forelimb clonus; Grade III: forelimb clonus; Grade II: rhythmic nodding; Grade I: tremor, facial muscle twitching, chewing; Grade 0: no seizures. A IV-V grade indicates a major epileptic seizure.

Each animal received an intraperitoneal injection of 100 mg/kg body weight of the S-phase marker BrdU (Solarbio Co., Ltd., Beijing, China) two days before the last PTZ injection. After the 8th PTZ injection, the mice were sacrificed with an anesthetic (1% barbital sodium, 50 mg/kg) 24 h after epilepsy.

### 4.2. LC-MS/MS Analysis for Neurotransmitter

The extraction of neurotransmitters was performed according to a previous study [39]. Briefly, mouse hippocampus weighing 10–20 mg was homogenized after the addition of 400 μL ice-cold methanol (0.1% formic acid). The homogenate was vortexed for 1 min and then centrifuged at 18,000× *g* for 15 min at 4 °C. The supernatant was transferred and evaporated to dryness under nitrogen. Then the dry residue was reconstituted in 100 μL of initial mobile phase (0.1% formic acid in water/methanol, 98:2, *v*/*v*), and 5 μL was injected into the LC-MS system.

The neurotransmitter analysis was conducted using an Acquity UHPLC/Q-Exactive-MS System (Thermo Fisher, Waltham, MA, USA) interfaced with a heated electrospray ionization (HESI) source and a hybrid quadrupole-orbitrap mass analyzer. A binary gradient system of 0.1% formic acid in water (solvent A) and methanol (solvent B) was utilized with an Acquity BEH C18 column (2.1 mm × 100 mm, 1.7 μm, Waters, Milford, MA, USA) at 35 °C and a flow rate of 0.4 mL/min with a gradient elution: 0 min, 98% B; 3–5 min, 20% B; 8 min, 98% B. The analyses were conducted in electrospray ionization (ESI) positive mode with sheath gas flow rate set at 60 L/min, aux gas flow rate at 10 L/min; sweep gas flow rate at 1 L/min, spray voltage at 2.75 kV, capillary temperature at 325 °C, and aux gas heater temperature at 400 °C. MS acquisition of Glu, GABA, DA, 5-HT, and 5-HIAA was performed in electrospray positive ionization parallel reaction monitoring (PRM) mode. The compound dependent parameters used for analysis are summarized in Table S1.

### 4.3. Histological Examination

The whole brain of the mice was carefully detached and fixed in paraformaldehyde overnight for further analysis. The brain tissues were embedded in paraffin and sliced into 5 μm coronal sections, and then stained for myelin with Luxol Fast Blue (LFB) and Nissl staining (Sigma, St. Louis, MO, USA) for the evaluation of CNS demyelination and Nissl body (Beyotime Institute of Biotechnology, China) to examine the neuronal morphology in the hippocampus using a microscope (Nikon/Ni-E, Tokyo, Japan).

### 4.4. Immunohistochemistry

For immunohistochemical staining, paraffin-embedded brain sections were deparaffinized and then incubated with 3% H_2_O_2_ for 15 min to remove the endogenous peroxidase activity. Subsequently, the sections were heated with a sodium citrate antigen retrieval solution for 15 min and blocked with non-immune goat serum for 30 min at 37 °C. Thereafter, the sections were washed with PBS and then incubated with a mouse anti-MBP antibody (1:1000; #78896; Cell Signaling Technology, Beverly, MA, USA) overnight at 4 °C. The next day, sections were washed with PBS three times and then incubated with a general secondary antibody (Epizyme; Shanghai, China) at room temperature for 1 h. The sections were performed with a DAB kit (Bioss; Beijing, China) for further analysis.

### 4.5. Immunofluorescence Detection of Brain

The sections from each group were used for immunofluorescence with GFAP and BrdU/DCX. For BrdU staining, sections were incubated with the following primary antibodies: mouse monoclonal anti-BrdU (1:1000, #5292, Cell Signaling Technology, USA) and rabbit polyclonal anti-DCX (1:2000, ab18723, Abcam, Cambridge, UK); polyclonal rabbit anti GFAP (1:200, #80788, Cell signaling technology, USA). Sections were then rinsed in PBS, followed by incubation with the following secondary antibodies: cyanine 3 goat anti rabbit IgG (GB21303, 1:500, Servicebio, Wuhan, China), cyanine 5 goat anti-rabbit IgG (GB27303, 1:500, Servicebio, China), and FITC goat anti-rabbit IgG (GB22303, 1:500, Servicebio, China) for one hour at room temperature. After washing, nuclei were counterstained with DAPI Fixed Cell Stain (Servicebio Co., Ltd., China). Sections were then mounted on slides and dehydrated. Finally, slides were coverslipped and kept in darkness at 40 °C for analysis.

### 4.6. Western Blot and ELISA Analysis

The total DNA/RNA/Protein Kit (R6734, Omega Bio-tek, Norcross, GA, USA) was utilized to extract total protein from hippocampal tissue according to the manufacturer’s instructions. Western blot was conducted for CREB (#9197, Cell signaling technology, USA), *p*-CREB (#9198, Cell signaling technology, USA), BDNF (ab108319, Abcam, UK), AKT (#9272, Cell signaling technology, USA), p-AKT (#4060, Cell signaling technology, USA), PSD95 (#3450, Cell signaling technology, USA), GABAA (GB113973, Servicebio, Wuhan, China), Synapsin-1 (SYN, #5297, Cell signaling technology, USA), Bax (#2772, Cell signaling technology, USA), Bcl-2 (#3498, Cell signaling technology, USA), Caspase-3 (#9662, Cell signaling technology, USA), Cleaved-caspase 3 (#9661, Cell signaling technology), NF-kB (P65) (#8242, Cell signaling technology, USA), NLRP3 (sc-134306, Santa Cruz, CA, USA), Caspase 9 (#9504, Cell signaling technology), Caspase 1 (#3866, Cell signaling technology), β-actin (bs-10966R, Bioss, Beijing, China) of hippocampal total protein. The density of protein bands was assessed using a computing densitometer. IL-1β was measured using ELISA kits (Nanjingjiancheng, Nanjing, China) according to the manufacturer’s protocols.

### 4.7. Fatty Acid Profile Analysis of Hippocampus

Hippocampus were homogenized with phosphate buffer to prepare the tissue homogenate. Total lipid was extracted from the homogenate using a modified method [40]. Briefly, the homogenate was extracted with chloroform: methanol 2:1 (*v*/*v*) and incubated for 1 h in a thermomixer. The mixture was then centrifuged at 10,000× *g*, 4 °C for 5 min. Deionized H_2_O was added to the supernatant before vortexing. After centrifugation, the lower organic phase was vacuumed to remove the solvent and obtain the total lipid.

A mixture of 37 fatty acid methyl ester (Sigma-Aldrich Inc., St. Louis, MO, USA) was used to perform qualitative analysis of fatty acids. Total lipid was dissolved and methylated with 2 mol/L hydrochloric acid in methanol for 1 h before injecting it into a gas chromatography Agilent 7820A (Agilent Technologies Inc., Shanghai, China) with a flame ionization detector. Nitrogen was used as a carrier gas. The analysis was carried out with a capillary column ATEO (30 m × 0.32 mm I.D and 0.25 μm film thickness; Lanzhou ATECH Technology Co., Ltd., Gansu, China). The temperatures of the injector and detector were 230 °C and 250 °C, respectively. The temperature of the oven started from 170 to 230 °C at a rate of 3 °C/min and ws retained at 230 °C for 20 min.

### 4.8. Statistical Analysis

The data were expressed as Mean ± SEM. Means comparisons between multiple groups were analyzed by one-way analysis of variance (ANOVA) analyzed by Tukey’s test, and *p* < 0.05 was considered statistically significant.

## 5. Conclusions

In summary, we demonstrated that dietary n-3 PUFA deficiency could affect resistance to PTZ-induced epilepsy, and the decline of n-3 PUFAs in the brain led to brain function disorders, including neurotransmitter imbalance, hippocampal neuronal apoptosis, demyelination, and neuroblasts, and inflammation, thus increasing the susceptibility to PTZ-induced seizures in adult offspring. Nutritional supplementation with n-3 PUFAs in adulthood could enhance tolerance to PTZ-induced seizures and alleviate epilepsy induced brain dysfunction. The amelioration effect of DHA-enriched phospholipid on brain dysfunction caused by epilepsy was superior to that of DHA-enriched ethyl ester, and α-LNA exhibited the least effect. Molecular structure characteristics and different supplementation efficiencies of DHA to the brain might account for the different effects. Further, nutritional status in early life should be vitally considered because postnatal supplements may not fully restore normal status in adulthood.

## Figures and Tables

**Table 1:** Effects of dietary n-3 PUFAs in adulthood on the fatty acid composition alteration in the hippocampus of mice fed n-3 PUFA deficiency in early life and adulthood.

Fatty Acid (%)	AM	DN	DM	ALA	EE-DHA	PL-DHA
C14:0	0.21 ± 0.09	0.54 ± 0.10	0.60 ± 0.15	0.46 ± 0.18	0.23 ± 0.10	0.65 ± 0.23
C14:1	2.32 ± 0.21	2.03 ± 0.15	2.01 ± 0.09	2.33 ± 0.42	2.12 ± 0.38	1.72 ± 0.35
C16:0	19.76 ± 1.09	19.56 ± 1.19	21.43 ± 1.08	19.87 ± 1.51	21.47 ± 1.91	19.44 ± 1.47
C16:1	0.81 ± 0.09	0.83 ± 0.15	0.95 ± 0.20	1.02 ± 0.10	0.72 ± 0.19	0.87 ± 0.13
C18:0	21.13 ± 1.24	21.91 ± 1.51	21.23 ± 0.92	22.04 ± 1.48	21.62 ± 1.75	20.55 ± 1.37
C18:1	20.42 ± 1.99	19.18 ± 2.08	19.83 ± 1.01	19.61 ± 0.91	20.13 ± 1.16	19.84 ± 1.31
C18:2 (n-6)	0.71 ± 0.21	0.32 ± 0.10	0.39 ± 0.11	0.43 ± 0.14	0.42 ± 0.21	0.39 ± 0.15
C20:1	3.19 ± 0.32 ^a^	2.28 ± 0.22 ^b^	2.74 ± 0.19 ^ab^	2.83 ± 0.22 ^ab^	2.08 ± 0.29 ^b^	2.56 ± 0.37 ^b^
C20:3 (n-3)	1.01 ± 0.09	1.24 ± 0.19	1.02 ± 0.31	0.36 ± 0.13	0.52 ± 0.15	1.23 ± 0.24
C20:4 (n-6)	8.52 ± 0.91 ^ab^	9.20 ± 0.58 ^a^	8.69 ± 0.61 ^ab^	7.83 ± 0.39 ^c^	7.55 ± 0.57 ^bc^	8.37 ± 0. 62 ^ab^
C22:4 (n-6)	2.77 ± 0.16 ^b^	3.49 ± 0.23 ^a^	3.72 ± 0.52 ^a^	3.35 ± 0.61 ^ab^	3.28 ± 0.42 ^ab^	3.11 ± 0.27 ^ab^
C22:5 (n-6)	0.44 ± 0.12 ^d^	7.97 ± 0.47 ^a^	6.75 ± 0.37 ^a^	4.61 ± 0.36 ^b^	3.84 ± 0.28 ^c^	3.75 ± 0.25 ^c^
C24:0	0.93 ± 0.10	0.77 ± 0.19	0.76 ± 0.15	1.17 ± 0.21	0.38 ± 0.09	0.97 ± 0.12
C22:6 (n-3)	14.85 ± 1.07 ^a^	8.52 ± 0.87 ^c^	7.41 ± 0.61 ^c^	11.05 ± 0.82 ^b^	12.83 ± 1.31 ^a^	13.61 ± 1.09 ^a^
Other fatty acid	2.93 ± 0.21	2.17 ± 0.19	2.47 ± 0.32	3.04 ± 0.28	2.82 ± 0.16	2.94 ± 0.35
∑n-6 LC-PUFA/ ∑n-3 LC-PUFA	0.78 ± 0.12 ^d^	2.43 ± 0.19 ^a^	2.04 ± 0.21 ^a^	1.35 ± 0.11 ^b^	1.06 ± 0.09 ^c^	1.11 ± 0.10 ^c^

Note: Data were presented as mean ± SEM. Different letters indicated significant difference at *p* < 0.05 among the six groups determined by ANOVA (Tukey’s test). ∑n-3 LC-PUFAs: data were calculated as C20:3 (n-3) + C22:6 (n-3). ∑n-6 LC-PUFAs: data were calculated as C18:2(n-6) + C20:4 (n-6) + C22:4 (n-6) + C22:5 (n-6).

## Data Availability

The data presented in this study are available on request from the corresponding author.

## Supplementary Materials

The following supporting information can be downloaded at: https://www.mdpi.com/article/10.3390/md21060354/s1; Figure S1: Body weight variation of the mice during PTZ administration; Table S1: parameters of neurotransmitters for MS condition.
