# Supplementary material for: Supplementation of n-3 PUFAs in Adulthood Attenuated Susceptibility to Pentylenetetrazol Induced Epilepsy in Mice Fed with n-3 PUFAs Deficient Diet in Early Life
Source: Mar Drugs. 2023 Jun 9;21(6):354. doi: 10.3390/md21060354 (PMC10305078; doi:10.3390/md21060354)
Supplement: Supplementary file 1 [file marinedrugs-21-00354-s001.zip › marinedrugs-2397476-supplementary.pdf]

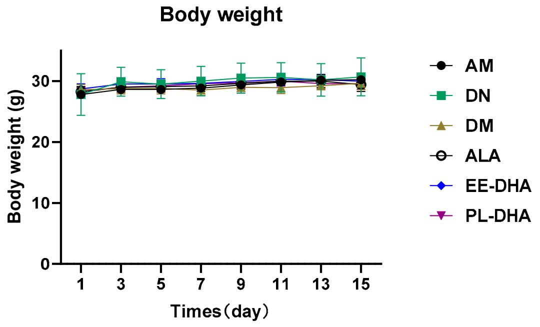

Figure S1 Body weight variation of the mice during PTZ administration.

Table S1 Parameters of neurotransmitter for MS condition.

Name	Abbreviation	Ion mode	precursor ion (m/z)	product ion (m/z)	NCE (V)
Glutamate	Glu	+	148	130	40
γ-aminobutyric acid	GABA	+	104.1	87	40
Dopamine	DA	+	154.1	137.1	40
5-hydroxytryptamine	5-HT	+	177.1	160.1	90
5-hydroxyindoleacetic acid	5-HIAA	+	192	146	80
